# Ligand Specificity of Group I Biotin Protein Ligase of *Mycobacterium tuberculosis*

**DOI:** 10.1371/journal.pone.0002320

**Published:** 2008-05-28

**Authors:** Sudha Purushothaman, Garima Gupta, Richa Srivastava, Vasanthakumar Ganga Ramu, Avadhesha Surolia

**Affiliations:** 1 Molecular Biophysics Unit, Indian Institute of Science, Bangalore, India; 2 National Institute of Immunology, Aruna Asaf Ali Marg, New Delhi, India; University of Arkansas, United States of America

## Abstract

**Background:**

Fatty acids are indispensable constituents of mycolic acids that impart toughness & permeability barrier to the cell envelope of *M. tuberculosis*. Biotin is an essential co-factor for acetyl-CoA carboxylase (ACC) the enzyme involved in the synthesis of malonyl-CoA, a committed precursor, needed for fatty acid synthesis. Biotin carboxyl carrier protein (BCCP) provides the co-factor for catalytic activity of ACC.

**Methodology/Principal Findings:**

BPL/BirA (Biotin Protein Ligase), and its substrate, biotin carboxyl carrier protein (BCCP) of *Mycobacterium tuberculosis* (*Mt*) were cloned and expressed in *E. coli* BL21. In contrast to *Ec*BirA and *Ph*BPL, the ∼29.5 kDa *Mt*BPL exists as a monomer in native, biotin and bio-5′AMP liganded forms. This was confirmed by molecular weight profiling by gel filtration on Superdex S-200 and Dynamic Light Scattering (DLS). Computational docking of biotin and bio-5′AMP to *Mt*BPL show that adenylation alters the contact residues for biotin. *Mt*BPL forms 11 H-bonds with biotin, relative to 35 with bio-5′AMP. Docking simulations also suggest that bio-5′AMP hydrogen bonds to the conserved ‘GRGRRG’ sequence but not biotin. The enzyme catalyzed transfer of biotin to BCCP was confirmed by incorporation of radioactive biotin and by Avidin blot. The K_m_ for BCCP was ∼5.2 µM and ∼420 nM for biotin. *Mt*BPL has low affinity (K_b_ = 1.06×10^−6^ M) for biotin relative to *Ec*BirA but their K_m_ are almost comparable suggesting that while the major function of *Mt*BPL is biotinylation of BCCP, tight binding of biotin/bio-5′AMP by *Ec*BirA is channeled for its repressor activity.

**Conclusions/Significance:**

These studies thus open up avenues for understanding the unique features of *Mt*BPL and the role it plays in biotin utilization in *M. tuberculosis.*

## Introduction

Tuberculosis still remains a major cause of death, especially in developing countries. The resurgence of this disease has primarily been due to the emergence of drug resistant tubercle, especially to the most effective drug, isoniazid (INH) (1–3). The ability of bacteria to survive inside hostile environment of host macrophage is primarily due to its complex lipid bilayer formed by mycolic acids, glycolipids, lipoproteins etc. In fact, it is estimated that lipids constitute 40% of mycobacterial dry cell weight. Thus, biosynthesis and assembly of mycolic acids and other lipids constitute potential targets for chemotherapeutic intervention for treating tuberculosis (4–6). *M.tuberculosis* genome contains ∼250 enzymes involved in fatty acid synthesis as compared to just about 50 in *E.coli* (7, 8). Fatty acid synthesis involves carboxylation of nascent chain which requires acetyl CoA carboxylase, a biotin dependent carboxylase which has three functionally dissimilar domains: (1) biotin carboxyl carrier protein (BCCP); (9) biotin carboxylase component that catalyzes Mg- ATP-dependent carboxylation of BCCP to form {COO}^−^- BCCP; and transcarboxylase component that catalyzes transfer of carboxyl group from {COO}^−^- BCCP to acetyl CoA to form malonyl CoA (9,10). ApoBCCP is biotinylated by **B**iotin **P**rotein **L**igase (BPL/BirA) to form holoBCCP, which then participates in carboxylation reaction. The biotinylation reaction by BPL is a two-step process:

Biotin+ATP→biotinyl-5′-AMP+PPi

Bio-5′-AMP+apocarboxylase (BCCP)→holocarboxylase (biotin- BCCP)+AMP

BPL (EC 6.3.4.15) catalyses transfer of biotin to an ε -amino group of a specific lysine residue, which is usually the 35^th^ amino acid from C-terminal of apoBCCP and converts it to active holoBCCP which promotes fatty acid initiation and elongation (11, 12). The biotinylation reaction is highly specific, as BCCP subunit of biotin dependent carboxylase is the only substrate for BPL.

There are two variants of prokaryotic BPLs classified as:

*Group I*, a monofunctional enzyme lacks N-terminal HTH domain e.g. *M.tuberculosis, A.aeolicus, P.horikoshii.*

*Group II*, a bifunctional enzyme, has N-terminus HTH domain which attributes repressor function to the protein e.g. *E.coli, B.subtilis, P.aeruginosa*.

In Group II BPL, adenylated biotin (bio-5′-AMP), an intermediate of BPL/BirA catalyzed reaction complexes with the protein (*E.coli* BirA – 321 amino acids) to cooperatively repress genes of biotin biosynthetic pathway (12). Group I BPL (*Mycobacterium tuberculosis* BPL – 266 amino acids) lacks N-terminal DNA binding domain and hence probably does not function as a repressor (13). So, the mechanism of repression of biotin biosynthetic pathway of group I BPL and organisms harboring them is yet to be ascertained.

One of the most successful anti tuberculosis drug, isoniazid, also inhibits elongation of fatty acid. Thus, our interest was focused on biotin protein ligase as it globally determines level of fatty acid synthesis. In this study, we expressed purified and characterized *M.tuberculosis* BPL (*Mt*BPL) and its substrate BCCP. Moreover, this is the first report on thermodynamic parameters of group I BPL.

## Results

**Figure 1:**
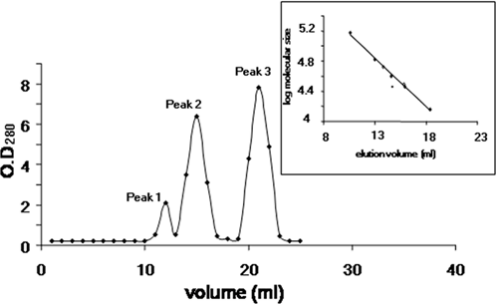
Molecular weight determination of BPL species by gel exclusion chromatography. BPL catalyzed reaction: 10 mM Tris-HCl p H-7.5 containing 5 nM BPL, 3 mM ATP, 5.5 mM MgCl_2_, 500 µM biotin but no BCCP were incubated at 37°C for 1 h. The reaction mixture was then loaded on to Superdex S 200. Peak 1: Dimeric BPL –bio 5′AMP-BPL (M_r_ ∼60,000±2900 Da). Peak 2: Monomeric BPL (M_r_ ∼29,500±150 Da). Peak 3: Unreacted ATP.

*Determination of dimerization of BPL by Superdex S200 column*: Equimolar concentration of purified i) BPL, ii) BPL catalysed reaction: 10 mM Tris-HCl, pH- 8.0 containing 0.1 mM BPL, 3 mM ATP, 5.5 mM MgCl_2_, 500 µM biotin and iii) negative control which is reaction mixture without BPL were incubated at 37°C for 1 h. The whole enzymatic process was done in the absence of BCCP, in order to determine the oligomeric status of adenylate intermediate of BPL. The reaction mixture was then individually loaded onto Superdex S-200 FPLC column. BPL without its substrate eluted as a single peak at volume corresponding to a Mr of ∼29,500±150 Da, confirming that BPL exists mostly as a monomer in its native state. This was further confirmed by Dynamic Light Scattering (DLS). *Mt*BPL incubated with biotin also eluted as a single peak of molecular weight of ∼29,500. *Mt*BPL catalyzed reaction with biotin and ATP resulting in bio-5′AMP, gave three peaks corresponding to a minor peak of molecular weight range Mr of 56,000±350 Da, a second peak of Mr ∼29,500±150 Da and a third peak corresponding to low molecular mass (Figure 1). The sample that eluted at lower molecular mass range (peak 3) displayed absorption in UV region and was confirmed as ATP by thin layer chromatography. The minor peak 1 corresponding to molecular weight range of Mr ∼56,000 Da was the dimeric *Mt*BPL - bio-5′AMP - *Mt*BPL. The second peak of molecular weight range Mr ∼29,500 was monomeric *Mt*BPL - bio-5′AMP. Both peak 1 and 2 were holoenzyme (*Mt*BPL - bio-5′AMP), as they biotinylated *Mt*BCCP in the absence of biotin and ATP. To confirm if the peaks exist in equilibria, the monomeric peak was repartitioned on the same column. The monomeric species eluted as a single peak of molecular size of ∼29,500 Da. This confirms that on catalysis *Mt*BPL forms monomeric and a weakly formed physiological dimer, both of which were holoenzymes. All the three species: apo-*Mt*BPL, *Mt*BPL – biotin and *Mt*BPL - bio-5′AMP were predominantly monomers as confirmed by DLS.

*Modeling the putative amino acid residue interactions with ligand atoms:*

Sequence alignment and crystal structure of *Pyrococcus horikoshii* BPL (*Ph*BPL), *Ec*BirA and *Mt*BPL show conserved residues that are critical for substrate binding needed for enzyme activity (Table 1). The three BPLs share function and sequence homology, but differ in their oligomerization state and ligand interaction. The ligand atoms were numbered by LPC/CSU software and the ordered structure is provided in Table S1.

**Table 1:** Conserved residues among BPLs of different species

	*SS1*	*SS2*	*SS3*	*SS4*
*Ph*BPL	^45^G**H**GRL***N***RKW ^53^	^100^KWPND^105^	^111^K **I**AG**V**LVE^118^	^124^G**I**GLN^128^
*Mt*BPL	^66^GRGRHGR***G***W ^74^	^127^KWPND^131^	^138^KLAGIL**A**E^143^	^52^G**V**GLN^156^
*Ec*BirA	^115^GRGRRGRKW^123^	^72^KWPND^176^	^183^ KLAGILVE^190^	^203^G**A**GIN^207^

The residues in bold are structurally similar amino acid and the residues in bold, italics are structurally unrelated amino acids.

**Figure 2:**
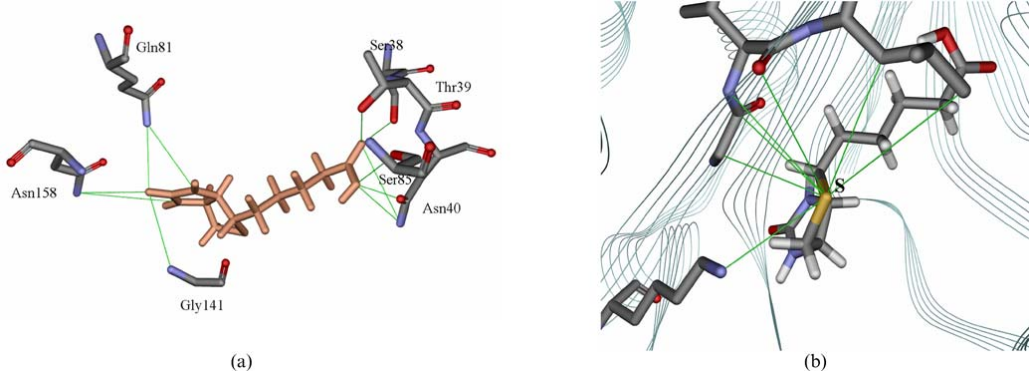
Hydrogen and hydrophobic interactions between biotin and *Mt*BPL. (a) The H- bond interactions between biotin and the amino acid residues of *Mt*BPL. The figure was generated using ViewerLite42. The contact of structural units (CSU) was derived with LPC software. The lines indicate H-bonds and the amino acid residues denote the interacting molecules. (b) Interaction of thiophene S atom of biotin with *Mt*BPL. The figure was generated using ViewerLite42. The contact of structural units (CSU) was derived with LPC software. The lines indicate H-bonds and the amino acid residues denote the interacting molecules.

Biotin: The association of biotin and *Mt*BPL is stabilized by 52 interactions, of which 11 are H- bonded 19 are hydrophobic interactions and 22 are other interaction *viz.*, salt bridge and van der waal's interactions. Residues Ser38, Thr39, Asn40, Gln81, Ser85, Gly141, Asn158 form strong hydrogen bonds with biotin. The N-terminal amino acids are involved in 8 hydrogen bonds with biotin. Leu143 and Gly156 make six and two hydrophobic interactions respectively with biotin. The biotin carboxylate hydrogen bonds with side chain oxygen atoms of Ser38 and Thr39 (Figure 2a). The ureido nitrogen forms hydrogen bond with Gln81, Asn158 and the thiophene ring ‘S’ makes hydrophobic contacts with Lys138, Gly141, Ile142, Leu143 (Figure 2b;Table 2,3). The conserved ‘GXGRRG’ sequence of *Ec*BirA and *Ph*BPL interacts with biotin as confirmed by crystal and experimental studies. However, no such hydrogen bond interactions were observed in *Mt*BPL - biotin association from computational analysis. The binding energy for association of biotin to *Mt*BPL by computational analysis is – 3.645 kcal/mol.

**Table 2:** List of putative hydrogen bonds between ligands biotin/bio-5′AMP and *Mt*BPL

Residues	Ligands	
	Biotin	Bio-5′AMP
Ser38 O^γ^	O16/**3.0**	-
Thr39 O^γ1^	O16/**3.1**	-
	-	-
Thr 39 N	-	-
Asn40 O^δ1^	O15/**2.3**	-
Asn40 N^ δ2^	O15/**3.0**	O23/**4.6**
		O35/**5.3**
Asn 40 N	O16/**2.9**	-
Gln63 N^ε2^	-	O23/**4.9**
	-	O35/**4.3**
Ala65 O	-	O35/**5.1**
Arg67 N	-	O23/**3.8**
His70 O	-	N24/**3.7**
His70 N^δ1^	-	N29/**3.5**
	-	N33/**3.3**
Gly71 O	-	N2/**5.0**
	-	O23/**4.5**
Arg72 O	-	N2/**5.1**
Gly73 N	-	N2/**4.4**
Trp74 O	-	N2/**3.4**
	-	N10/**3.3**
Trp74	-	O13/**3.7**
Gln81 N^ε2^	O13/**3.5**	-
	N2/**4.1**	-
Ser85 O^γ^	O15/**3.4**	-
Asn130 N^δ.2^	-	N29/**3.5**
	-	N33/**4.7**
Asn130 N	-	N29/**4.1**
Asp131 N	-	N29/**4.1**
	-	N30/**4.0**
Asp131 O^δ1^	-	N29/**4.1**
	-	N30/**3.0**
Gly141 N	O13/**4.2**	O37/**3.4**
Ile142 O	-	N26/**3.7**
	-	N30/**3.5**
Ile142 N	-	N26/**3.8**
Ile142 N	-	N30/**3.7**
Gly156 O	-	O37/**3.0**
Asn158 N^δ2^	N10/**5.3**	O36/**4.4**
	O13/**3.7**	O37/**4.4**
Glu164 O^ε1^	-	036/**5.1**
Glu165 O^ε2^	-	N10/**3.6**
Asp261 O^δ1^	-	N29/**4.4**

**Table 3:** Type of contacts made by biotin / bio-5′AMP with the *Mt*BPL

Type of bonds	Biotin	Bio-5′AMP
Hydrogen	11	35
Hydrophobic	19	27
Acceptor-Acceptor	3	4

**Figure 3:**
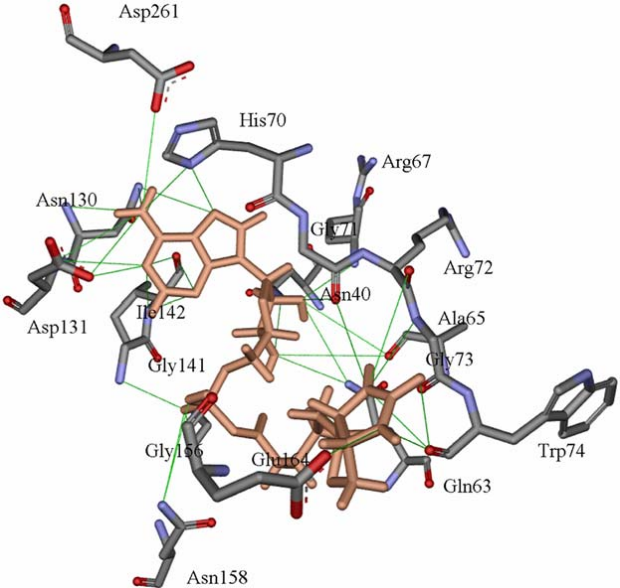
The H- bond interactions between bio-5′AMP and the amino acid residues of *Mt*BPL. The H- bond interactions between biotin and the amino acid residues of *Mt*BPL. The figure was generated using ViewerLite42. The contact of structural units (CSU) was derived with LPC software. The lines indicate H-bonds and the amino acid residues denote the interacting molecules.

Bio-5′AMP: The association of bio-5′AMP to *Mt*BPL is strengthened by 35 H-bonds relative to only 11 H-bonds with biotin, 27 hydrophobic interactions and 55 other weak interaction. On formation of bio-5′AMP, the interacting atoms are altered. The N-terminal amino acids Ser38, Thr39 are no longer involved in stabilization of *Mt*BPL – bio-5′AMP complex. However, residues Gln63-Trp74 of the conserved ‘GRGRHGR’ form H-bond interaction with bio-5′AMP. Trp74 which is a conserved residue interacts with bio-5′AMP via H-bond. The ureido nitrogen interacts via H-bond with Gly71, Arg72, Gly73, and Trp74. The thiophene ‘S’ now forms strong hydrophobic interaction with Ala75, Ala76, Gln81, Ala163, Pro163. The adenine ring forms three hydrogen bonds each with His70, Asn130, Asp131, and ILe142 (Figure 3; Table 2,3). The binding energy for bio-5′AMP association to *Mt*BPL by computational analysis is – 3.120 kcal/mol.

**Figure 4:**
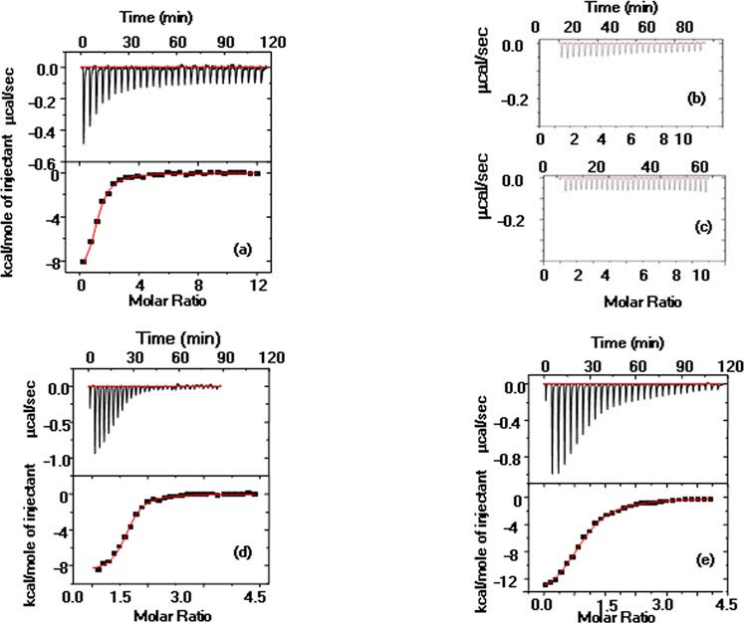
Isothermal calorimetry traces of the interaction of *Mt*BPL with its ligands. These experiments were performed in standard buffer – 10 mM Tris-HCl pH-7.5, 200 mM KCl, 2.5 mM MgCl_2_.Twenty nine 10 µl aliquots from a 100 µM solution of (a) biotin (b) desthiobiotin (c) ATP *were* used for the titrations. The lower panel shows the least squares fit of the data in the upper panel in (a) to obtain the binding parameters of the interactions of *Mt*BPL with biotin.The fit of the data is not provided for reactions where interaction between *Mt*BPL and a given ligand was not observed viz (b) desthiobiotin or (c) ATP. (d) *Mt*BPL was incubated with 100 µM biotin for 30 min in the ITC cell and then titrated against twenty nine injections of 10 µl aliquots from 100 µM ATP solution. (e) Twenty nine injections of 10 µl each from a 50 µM solution of bio-5′AMP were injected into 5 µM *Mt*BPL. The lower panel shows the least squares fit of the data in the upper panels of (d) and (e) to obtain the binding parameters of the interactions of *Mt*BPL with biotin + ATP and Bio-5′-AMP respectively.

Desthiobiotin: Simulated docking of desthiobiotin to *Mt*BPL show 16 H-bond and 30 hydrophobic interactions. The major difference between biotin and desthiobiotin (DTB) is the absence of thiophene ring in DTB. Hence, the contact between the thiophene ring and the protein is lacking in the analog. When the relative strengths of the bonds were analyzed, it was observed that the number of strong hydrogen bonds (donor- acceptor distance *D –A*<3.0 Å) were four for biotin and just one for desthiobiotin. These two factors probably contribute to the lack of desthiobiotin association with *Mt*BPL (Figure 4b) in our experimental studies. And indeed the association of desthiobiotin to *Mt*BPL by computational docking is unfavorable with binding energy of +0.984 kcal/mol.

*Isothermal calorimetry:* The binding affinity of BPL for various ligands: biotin, bio-5′-AMP, desthiobiotin, ATP, ADP and AMP was determined by calorimetric titration. A typical ITC trace for binding of biotin to BPL is shown in Figure 4a. The free energy for binding of biotin to *Mt*BPL was −8.08 kcal/mol whereas association of *Ec*BirA-biotin is −10 kcal/mol. Biotin binds to *Mt*BPL at a single site with high affinity of 1.06×10^−6^ M, a value that was less compared to that of *E.coli* BirA (4.2×10^−8 ^M) but four fold greater than the K_d_ for biotin binding to N-terminal deleted *Ec*BirA (65–321). *Mt*BPL had almost similar affinities for biotin, bio-5′-AMP and biotin- ATP binary substrates. The ligands: ATP, AMP, ADP and desthiobiotin did not show any measurable enthalpy changes on association with *Mt*BPL. Since desthiobiotin is a precursor of biotin, affinity of *Mt*BPL for this desthio molecule was also determined. Earlier reports suggest that *Ec*BirA can non-selectively incorporate desthiobiotin into BCCP at higher concentration. However in *Mt*BPL, desthiobiotin association did not show appreciable heat changes even at the highest ligand concentration (1.25 mM) tested. The calorimetric titration with desthiobiotin was also performed at 2.5 fold higher enzyme concentration. Figure 4b shows the ITC trace for titration of 100 µM desthiobiotin against 10 µM *Mt*BPL. Thus inability of *Mt*BPL to incorporate desthiobiotin in biotinylation reaction was due to its lack of affinity for desthiobiotin.

ATP, the other substrate of *Mt*BPL did not show appreciable heat change in calorimetric titration (Figure 4c). However, when *Mt*BPL saturated with biotin was titrated with ATP, its dissociation constant was 1.16×10^−6^ M (Figure 4d). Thus, biotin binding to *Mt*BPL opens site for binding of ATP on the protein. Bio-5′-AMP, an intermediate of enzyme catalyzed reaction was titrated against BPL. The dissociation constant for bio-5′-AMP was 1.36×10^−6^ M (Figure 4e), which was almost similar to that of *Mt*BPL- biotin association. This is in contrast to that observed in *Ec*BirA which had a 1000 fold greater affinity for bio-5′-AMP over biotin. The thermodynamic parameters governing binding of biotin to *Mt*BPL are provided in Table 4.

**Table 4:** Thermodynamic parameters governing effector binding to *Mycobacterium tuberculosis* BPL

Ligands	n	K_b_×10^6^ (M^−1^)	ΔH (cal/mol)	ΔG (kcal/mol)	ΔS (cal/mol/K)
**Biotin**	0.9814	1.06	−11.12	−8.087	−10.35
**Biotin+ATP**	0.9229	1.16	−8.18	−9.07	−2.925
**Bio-5′AMP**	0.9800	1.36	−17.25	−7.97	−31.7

Titrations were carried out in 10 mM Tris-HCl, ph-7.5 containing 200 mM KCl, 2.5 mM MgCl_2_ using a VP-ITC calorimeter (Microcal, Inc.,USA). Binding parameters K_b_ and ΔH were determined b fitting of ITC data to a single-site binding model in the Origin software package. Binding free energies (ΔG) and entropies (ΔS) were calculated using the equation, ΔG = RT *ln*K_b_ and ΔG = ΔH−TΔS. The values given above represent results of a single titration with each ligand and in multiple titrations the values of K_b_ and ΔH are reproducible within 2-fold and 1 kcal/mol.

**Figure 5:**
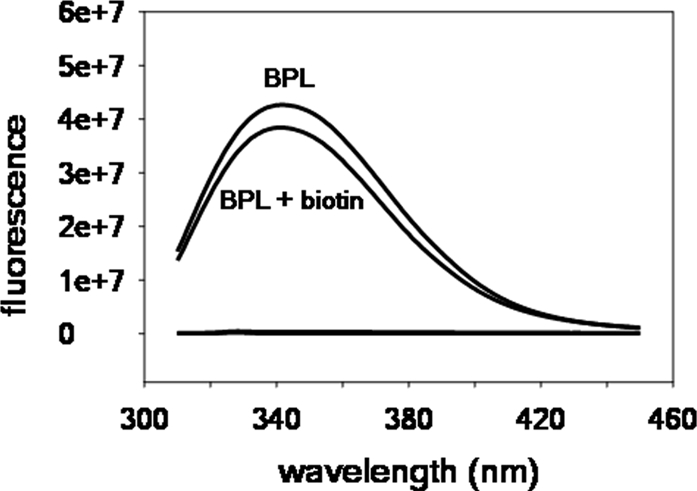
Flourescence titration. Steady state fluorescence emission spectra of MtBPL in absence and presence of biotin. [BPL] = 2 µM, [biotin] = 50 µM. All spectra were obtained in standard buffer at 20°C.

*Fluorescence titration of BPL*: Figure 5 shows steady state fluorescence emission spectra of *Mt*BPL, *Mt*BPL + biotin. The emission maximum for *Mt*BPL was at 342 nm similar to *Ec*BirA. The binding of biotin to the protein resulted in 10% quenching of intrinsic fluorescence signal.

*Biological properties of BPL*: The enzymatic activity of BPL was determined by incorporation of ^14^C labelled biotin into the cloned BCCP domain of *M.tuberculosis* acetyl CoA carboxylase. Optimal enzyme activity was observed at pH 7.5–8.0. The presence of biotin, ATP, Magnesium ions, apo-BCCP was necessary for biotinylation reaction. Enzyme activity was dependent on ATP as nucleotide source and substitution of ATP with GTP did not result in biotinylation reaction. BPL forms a weak dimer with bio-5′-AMP synthesized from ATP and biotin, in absence of apoBCCP as observed from gel filtration (Figure 1). The K_m_ for D-biotin is ∼424.2±56.38 nM, 21.08±3.78 µM for Mg/ATP, 5.2±.56 µM for apoBCCP and 10 nM of BPL was sufficient to drive enzymatic process (Table 5). These data thus confirm that cloned BPL of *M.tuberculosis* was enzymatically active.

**Table 5:** Kinetic constants for the interaction of *Mt* BPL with its substrates- biotin, MgATP and *Mt*BCCP

Substrate	K_m_ (µM)	k_cat_ (s^−1^)	k_cat_/K_m_ (s^−1^M^−1^)×10^3^
**Biotin**	0.420±.056	0.034±.007	82±2.1
**Mg/ATP**	21.08±3.78	0.0282±.003	1.33±.09
**BCCP**	5.2±0.56	0.030±.001	5.77±.37

The experiment was carried out in triplicates as described in Experimental procedures.

**Figure 6:**
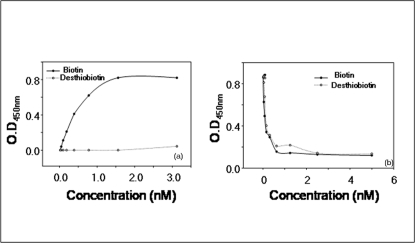
Enzyme linked immunosorbent assay to determine the substrate specificity of *Mt*BPL. (a) Incorporation of biotin / desthiobiotin into BCCP in BPL catalyzed reaction. The wells were coated with 15 µM BCCP and incubated overnight at 4°C. The non-specific sites were blocked with 1% BSA and biotinylation reaction carried out with biotin/desthiobiotin as the substrate. The incorporated substrate was detected streptavidin-HRP. All experiments were done in triplicates and on three different occasions. (b) Competitive ELISA to determine the affinity of streptavidin for biotin and desthiobiotin. The wells were immobilized with biotinylated BCCP. Streptavidin HRP (1∶2500) was incubated with equal volume and different concentration of biotin or desthiobiotin for 1 h at 37°C. The reaction mixture was then added to biotinylated BCCP. The unreacted streptavidin bound to immobilized BCCP which was detected by TMB/H_2_O_2_. All experiments were done in triplicates and at three different occasions.

*Specific incorporation of biotin by Mt*BPL: To determine if BPL can non-specifically utilize desthiobiotin in the enzyme reaction, both biotin and its analogue were used at concentrations ranging from 25 µM – 6 nM in biotinylation of BCCP (15 µM) by 10 nM of BPL. As revealed by an assay akin to ELISA, biotin at ∼620 nM efficiently biotinylated BCCP whereas desthiobiotin was not incorporated into BCCP in a 30 min biotinylation reaction (Figure 6a). The desthiobiotin concentration was increased to 1000 µM which was >1200 fold the optimal biotin concentration and the incubation period for 4 h. But the incorporation of desthiobiotin into BCCP by BPL was not appreciable. This could be due to two reasons: i) desthiobiotin was not utilized as a substrate in enzymatic process by BPL or, ii) streptavidin HRP was unable to detect incorporated desthiobiotin. To rule out the second possibility, a competitive assay of streptavidin with biotin/desthiobiotin was performed. Streptavidin HRP at 1∶2500 dilution of 1 mg/ml solution was pre-incubated with biotin/ desthiobiotin (5 µM–5 nM) for 1 h at 37°C and the solution was then added to previously immobilized biotinylated BCCP. Streptavidin interacts with its cognate molecule (biotin/desthiobiotin) and any unreacted, free streptavidin from first step will react with biotinylated BCCP determining affinity of streptavidin for biotin/desthiobiotin. Figure 6b shows that streptavidin interacts efficiently with both biotin and desthiobiotin. These experiments thus rule out the possibility that discrepancy in incorporation of biotin/desthiobiotin in BCCP was not due to error in detection of desthiobiotinylated BCCP but because of its non-utilization.

**Figure 7:**
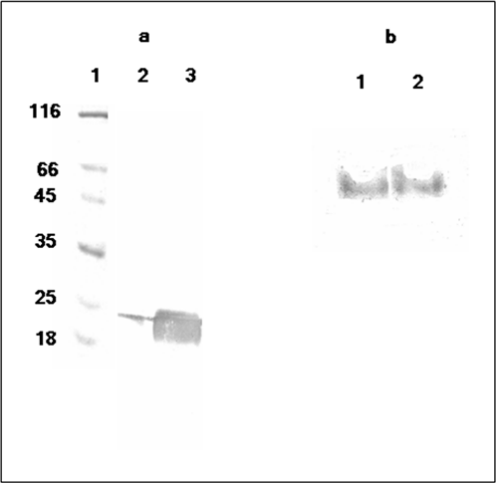
Avidin blot of biotinylated BCCP catalysed by BPL. (a) Biotinylation by BPL was carried in a reaction mixture containing 50 mM Tris pH-8.0, 3 mM ATP, 5 µM biotin, 5.5 mM MgCl_2_, 0.1 mM dithiothreitol, 0.1 mg/ml bovine serum albumin and 5 nM BPL and 15 µM BCCP over night at 4°C. The reaction mixture was then resolved on a 12% SDS PAGE and transferred to PVDF membrane. The membrane was then incubated with streptavidin HRP for 1 h at room temperature and developed with AEC/ H_2_O_2._ Lane 1: Molecular weight marker. Lane 2: *Mt*BCCP was incubated with biotin, ATP and MgCl_2_ but no *Mt*BPL. The band observed was due to biotinylation of BCCP by endogenous host BPL. Lane 3: *Mt*BPL catalysed biotinylation of BCCP. The intensity of the band was several folds higher than that of biotinylation by endogenous host BPL. (b) holo*Mt*BPL catalyzed reaction was performed in the absence of biotin and ATP. *Mt*BCCP was incubated with sample (peak 1, 2) independently in the absence of biotin and ATP for 30 min at 37°C and resolved on a 12% SDS-PAGE and transferred to PVDF membrane. Holo-*Mt*BPL (MtbPL-bio-5′AMP) transfers biotin to BCCP which was detected by streptavidin –HRP. Lane 1: Peak 1, Superdex S200. Lane 2: Peak 2, Superdex S200.

*Avidin blot:* Biotinylation of apo-BCCP, a biotin acceptor protein was detected by streptavidin HRP on Avidin blot (Figure 7a). Lane 2 is BCCP alone with no BPL. The faint band observed corresponds to BCCP that was probably biotinylated by endogenous *Ec*BirA. Lane 3 is biotinylation reaction of BCCP by BPL in presence of ATP, biotin and MgCl_2_.for 30 min at 37°C. As observed BCCP was biotinylated several fold by BPL in comparison with BCCP control (Lane 3), thus confirming transfer of biotin to acceptor domain of BCCP by *M.tuberculosis* BPL. Figure 7b shows the ability of both monomeric and dimeric *Mt*BPL associated with bio-5′AMP to biotinylate apoBCCP in the absence of biotin and ATP. The second step of the biotinylation reaction, the transfer of biotin to BCCP from bio-5′AMP by *Mt*BPL was detected by Avidin blot (Figure 7b). Lane 1 and 2 is the peak 1 and 2 (Figure 1) of holoenzyme eluted from gel exclusion chromatography biotinylating apoBCCP.

## Discussion

Acetyl CoA carboxylase and pyruvate dehydrogenase are enzymes crucial for fatty acid biosynthesis which require biotinylation of their BCCP domain as a prelude to their enzymatic activities (14, 15). Biotin protein ligase which globally controls this pathway is a potential target for anti-mycobacterial drugs. *Mt*BPL shares ∼25% and 31% sequence homology with *Ph*BPL and *Ec*BirA respectively. It belongs to monofunctional group I BPL as it lacks the 60 amino acid N-terminal HTH domain. This domain in *Ec*BirA contributes to repressor function by binding to biotin operon (bioO). Deletion of the N-terminal domain converted *Ec*BirA from bifunctional to monofunctional protein (16).

**Figure 8:**
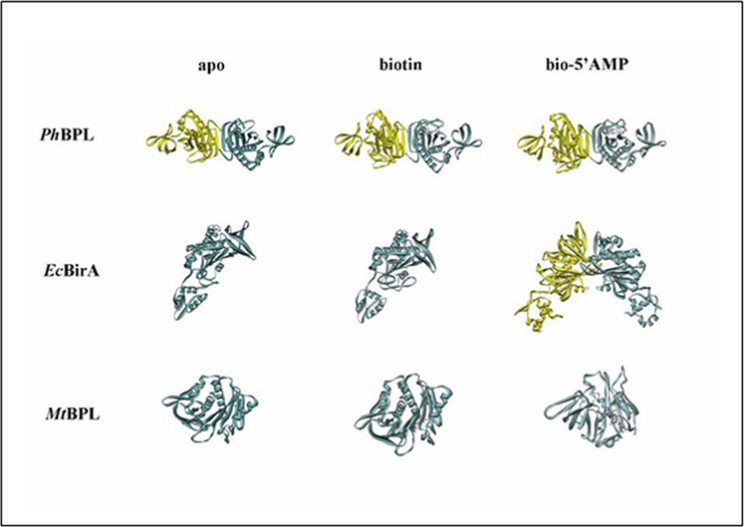
A schematic illustration of apo form, biotin and bio-5′AMP forms of *Ph*BPL, *Mt*BPL and *Ec*BirA.

The present study shows that *Mt*BPL differs from biotin ligase of other species both in interaction with its substrates and molecular mechanism of catalysis. Homodimerization is coupled to co- repressor synthesis and is a pre-requisite for repressor function in *Ec*BirA (16). Monofunctional *Ph*BPL (Group I) is a dimer in all the three forms viz. apo, biotin and bio-5′AMP liganded forms (Figure 8). *Ph*BPL in spite of lacking repressor function does exist as a dimer. Our studies reveal that apo, biotin and bio-5′AMP liganded. *Mt*BPL are monomers (Figure 1, 8). The peak corresponding to monomeric *Mt*BPL (peak 2, Figure 1) was holoenzyme as it biotinylated *Mt*BCCP. However, bio-5′AMP binding does promote weak physiological dimerization of *Mt*BPL similar to that observed when biotin binds to *Ec*BirA. But the ability of both peak 1 and 2 to transfer biotin to *Mt*BCCP confirmed that both the peaks were holoenzymes. Truncated *Ec*BirA lacks repressor function but is enzymatically active (bio-5′AMP synthesis). It would be interesting to study the oligomerization property of this truncated protein on bio-5′AMP synthesis Thus, our gel exclusion profiling studies suggest that monofunctional *Mt*BPL does not require dimerization for catalysis. Hence from studies on different BPLs, it is evident that oligomerization may not be a universal phenomena for all BPLs and some of them such as *Ph*BPL and *Ec*BirA, undergo oligomerization for compaction and thermo-stability in their apo/holo forms (17, 18).

It is known that *Ph*BPL is a dimer in all the three forms, apo, biotin and bio-5′AMP liganded, while *Ec*BirA is a dimer when associated with bio-5′AMP but monomeric in apo and biotin-liganded form. From our studies it was observed that *Mt*BPL is monomeric in all the three forms (Figure 8).

Sequence analysis shows conserved sequences between *Mt*BPL, *Ec*BirA and *Ph*BPL. Four homologous sequences between the three BPLs are shown (19). However, the identity of other residues dispersed in the protein sequences are also conserved but are not specified. Sequence SS1 interacts with biotin in *Ph*BPL and *Ec*BirA. Earlier studies by Kwon and Beckett (2000) show that the consensus ‘GRGRXGR’ sequence is involved in binding of biotin and bio-5′AMP (20). Docking studies of *Mt*BPL and biotin show that sequence SS1 was involved in interaction with bio-5′AMP but not biotin. In fact, except for residues ^66^Gly and ^67^Arg which were involved in hydrophobic interaction with the thiophene ring, the reminder of the conserved sequence appears to be of little or no consequence for the recognition of biotin. From ITC studies, it was observed that *Mt*BPL has a dissociation constant of 1×10^6^ M^−1^ for biotin (Figure 4a). Hence, the hydrogen bond between the hydrophilic residues, Ser 38, Thr39, Asn40, Gln81, Ser85 and biotin contribute to this association. The hydrophobic pocket formed by Ile83, Leu84, Ala140, Gly141, Ile142, Leu143, Gly154, Val 155, and Gly156 probably dock the thiophene ring of biotin. Bio-5′AMP interacts with Arg67, His70, Gly71, Arg72 of the conserved ‘GRGRHGR’ sequence by H-bonds and hydrophobic interaction. Asn158 contributes two H-bonds to the adenylate and the purine ring stacks on the face of the indole ring of Trp74 and makes H-bond with Asn158, and side-chain of alanine 65. An analogous interaction in *Ec*BirA, is provided by Phe which replaces alanine of *Mt*BPL at the homologous position. In *Ec*BirA, biotin binding site is a hydrophobic groove with hydrogen binding partners Ser89 and Thr90 pre- positioned to accept biotin (21). From our computational studies, it was observed that homologous residues Ser38 and Thr39 of *Mt*BPL H-bond with biotin. Thus, residues Ser38, Thr39 for biotin and ‘GRGRHGRGW’ sequence for bio-5′AMP may contribute largely to association of ligands. From docking simulations, biotin has 52 and bio-5′AMP has 117 interactions with *Mt*BPL. The number of interactions involved and the larger surface area occupied by bio-5′AMP protects *Ec*BirA from proteolysis. This suggests that though bio-5′AMP is an intermediate molecule; its complex with BPL is most stable. However, these computational analysis need to be confirmed by experimental studies.

The other focus of our study is the substrate specificity of *Mt*BPL for biotin. Desthiobiotin did not bind to *Mt*BPL nor was used in the biotinylation reaction. The thiophene ring and the hydrophobic tail would interact with the hydrophobic amino acids and the biotin carboxyl and ureido ring would interact with the hydrophilic amino acids. Since DTB lacks the thiophene ring, the interaction of the molecule with the hydrophobic amino acids is only via the hydrophobic tail and ureido ring. Also, biotin forms relatively stronger H-bonds (bond length >3.0Å) as compared to desthiobiotin. This is reflected in the unfavorable binding energy of association between desthiobiotin and *Mt*BPL (Biotin = −3.645 kcal/mol, Desthiobiotin = +0.984 l/mol).

A second conserved sequence of KWPNDVL shares sequence homology with biotin binding protein avidin. However, this sequence does not interact with the ligands in all the three BPLs. Lys111 (*Ph*BPL) and Lys183 (*Ec*BirA) of the conserved ‘KXAGXLVE’ play a critical role in adenylation reaction. However, from computational studies, no such interaction between this conserved lysine residue and the ligands were observed (21, 22).

Effector binding is central to a number of essential activation processes and provides essential information on interactions involved therein. The affinity of *Mt*BPL for its effectors biotin and bio-5′-AMP was determined by ITC. The thermodynamic data are categorized into enthalpy, free energy changes and dissociation constants. *Mt*BPL exhibited similar dissociation constants of ∼1×10^−6^ M for both the effectors (Figure 4a, d and e). In contrast, *Ec*BirA displayed different affinities for its ligands: biotin and bio-5′-AMP. Dissociation constants for biotin (4.2×10^−8^ M) and bio-5′AMP (4×10^−11^ M) was very different and hence they have been classified as ‘weak’ and ‘strong’ effectors respectively (23–25). The affinity of *Mt*BPL for bio-5′-AMP was 5 orders of magnitude lower than that observed in *Ec*BirA (4×10^−11^ M). Repressor function in *Ec*BirA is in association with bio-5′-AMP (co-repressor) and hence very tight binding is required to perform this function (26). However, the N-terminal deleted *Ec*BirA had 1000 fold reduced affinity (∼4×10^−8^ M) for bio-5′-AMP (16). This suggests that mono-functional group I BPLs exhibit attenuated affinities dedicated to perform only enzymatic function. The adenosine molecules, ATP, ADP and AMP did not show any appreciable heat changes in ITC. This is in contrast to crystallographic structure of *Ph*BPL which show interaction with adenosine molecules (22). However, in crystallographic structures, observations of the complexes of ligand which have extremely low affinities (<50 M^−1^) can often be observed which is typically difficult in biophysical studies. The endoplasmic reticulum chaperone, calreticulin, for example, does not show binding to glucose, though density for glucose complexed to calnexin, a very closely related molecule was seen in the crystal structure (27).

Bio-5′AMP, an intermediate of *Mt*BPL catalyzed reaction involves biotin and ATP as substrates. Hence, it was essential to determine the mechanism of association of *Mt*BPL with its substrates. These studies show sequential binding pattern of *Mt*BPL to them, as ATP (Figure 4c) does not interact with BPL directly but on saturation with biotin, it exhibits a K_d_ of ∼1.16×10^6^ M (Figure 4d). *Saccharomyces cerevisiae* BPL interacts first with ATP followed by biotin, in contrast to *Mt*BPL and *Ec*BirA (28). Biotin is the limiting substrate in *Mt*BPL catalyzed reaction similar to *Ec*BirA. Biotin binding perhaps contributes to structural transitions that may be required for interaction of ATP to form ternary complex between *Mt*BPL, biotin and ATP.

The Gibbs free energy for binding of biotin and bio-5′-AMP to *Mt*BPL were almost similar. But for *Ec*BirA, there was a gain of −4 kcal/mol of free energy on bio-5′-AMP binding. This is probably to ensure that repressor was always in active form for binding to *bioO* (29, 30). *Mt*BPL does not require any enhanced BPL-bio-5′-AMP stability for transfer of biotin to apoBCCP. Thus, thermodynamic studies reveal that molecular mechanisms of ligand interaction are very different between *Mt*BPL and *Ec*BirA on account of additional burden of repressor function in the latter molecule.

Binding of bio-5′-AMP to *Mt*BPL is associated with larger enthalpy changes (−17.2 kcal/mol) in comparison with −11.12 kcal/mol for biotin (Table 4). However in *Ec*BirA, enthalpy changes are larger for biotin (−19.2 kcal/mol) compared to that for bio-5′-AMP (-12.3 kcal/mol). According to Brown and Beckett (24), the absence of a charged functional group to interact with the adenosine moiety could be the reason for the enthalpic penalty. In *Ec*BirA, there is a biotin binding loop (BBL) 115–120 residues which is conserved and adenylate binding loop (ABL) consisting of residues 212–223 (24, 25). On limited proteolysis, the region between amino acid residues 217–218 was protected by bio-5′-AMP (26). This suggests that adenosine of bio-5′-AMP interacts at the ABL. BBL becomes ordered on biotin binding and then adenylate binding site is formed in *Ec*BirA. In contrast, there is no separate ABL and BBL in *Ph*BPL hence the biotin moiety of biotinyl-5′-AMP and biotin share the same binding site. Also, the adenosyl moiety of bio-5′-AMP and ADP interact almost at the same position. This sharing of binding site in *Ph*BPL which is strikingly different from that observed in *Ec*BirA suggests that catalytic binding sites are organized such that major conformational changes are perhaps not required in group I BPLs during the reaction (22). Bagautdinov *et al* (22) co-crystallized *Ph*BPL with biotin and bio-5′AMP suggesting that the interaction does not involve any conformational change. On the other hand, exposure of *Ec*BirA crystals to bio-5′-AMP resulted in cracking and crystal destruction due to conformational changes. Together, these data underscore striking differences in the organization of biotin and ATP binding site between *Mt*BPL and *Ec*BirA as well as the differences in the conformational changes they undergo upon binding of biotin/bio-5′-AMP.

The dissociation constant of *Mt*BPL for its ligands was high relative to *Ec*BirA (Table 4, Figure 4), hence enzyme turnover was determined by steady state kinetics. The kinetic constants show low K_m_ for both biotin (0.42 µM) and ATP (21 µM). This is similar to that observed in eukaryotic biotin auxotrophs such as *Saccharomyces cerevisiae* and *A.thaliana* which exhibit K_m_ in nanomolar range for biotin and lower micromolar concentration for MgATP (28, 32). *Ec*BirA has comparable K_m_ of 0.3 µM for biotin, but has higher K_m_ of 300 µM for ATP. Though *Mt*BPL has higher dissociation constant for both biotin and bio-5′-AMP, the K_m_ values were comparable to that of *Ec*BirA (Table 4,5). Thus, the lower binding affinity of *Mt*BPL for its ligands did not alter its enzymatic function.

Desthiobiotin (DTB) is precursor molecule for biotin but differs from it in several ways; both have an ureido ring but desthiobiotin lacks thiophene ring (Table S1). Wu *et al* (33) had suggested that *Ec*BirA incorporates desthiobiotin into SAPK, a protein with a biotinable lysine residue. Their assay was driven to 100% incorporation at a concentration of 1 mM in 4 h. But *Mt*BPL did not promote desthiobiotin incorporation into BCCP at increased concentration and incubation time (Figure 6a,b). From structural analysis of *Ph*BPL, it is evident that both thiophene and ureido side chains are accommodated in the hydrophilic bottom provided by glycine residues (Gly45,47,127,129) and the thiophene moiety interacts with the hydrophobic wall (22). DTB which lacks the thiophene side chain will not fit efficiently in such a structural frame work. The hexanoic acid attached close to ureido nitrogen may hamper interaction of ureido side chain as well. This argument is strengthened by the binding energy of *Mt*BPL (+0.984 kcal/mol) and *Ec*BirA (−1.897 kcal/mol) for desthiobiotin by computational modeling. Monofunctional *Mt*BPL has an unfavorable binding energy for the association and this is confirmed by the inability of the enzyme to either bind or incorporate desthiobiotin. *Ec*BirA prefers biotin (−3.491 kcal/ mol) to desthiobiotin (−1.897 kcal/mol) and hence utilizes desthiobiotin at higher molar concentration and increased incubation period. Biotin synthase (*BioB*) utilizes desthiobiotin to synthesize biotin and activity of the enzyme is determined by bioassay. This is a very tedious assay and there is a need for more reliable, easy, quantitative and rapid assay. Biotin synthase enzymatically generates one molecule of biotin from desthiobiotin. At equimolar concentration of biotin/desthiobiotin, *Mt*BPL preferably incorporates biotin and this can be exploited to assay biotin synthase activity in a sensitive manner.

In summary, we have successfully cloned an enzymatically active *Mt*BPL and biotin acceptor molecule, BCCP of *M. tuberculosis*. Our protocol variation of reduced growth and induction time yielded nearly 90% apo-BCCP. Our studies suggest that the mechanism of enzymatic process is at considerable variance from Group II BPL. *Mt*BPL binds initially to biotin with a moderate affinity as compared to *Ec*BirA. This complex then interacts with ATP to synthesize intermediate molecule bio-5′-AMP and biotin is transferred efficiently to BCCP. From our studies, it is clear that reduced affinity of *Mt*BPL does not alter enzyme kinetic parameters, suggesting that enhanced binding affinity in *Ec*BirA probably contributes to repressor activity. However, in this study we report that *Mt*BPL is merely not an equivalent of N-terminal deleted *Ec*BirA, but has its own unique *modus operandi*, including specificity and affinity for its ligands to promote biotinylation of BCCP domain of acetyl CoA carboxylase, an enzyme critical for survival of the pathogen. Also, the differences in the architecture of biotin and ATP binding site of *Mt*BPL and *Ec*BirA and the exquisite specificity of *Mt*BPL for biotin over desthiobiotin can be harnessed for synthesizing inhibitors for this important enzyme which should aid in combating global spread of tuberculosis.

## Materials and Methods

### Biochemicals

D-biotin, desthiobiotin, IPTG and ATP (disodium salt) were purchased from Sigma, USA. Restriction enzymes were obtained from MBI Fermentas, USA. Ni-NTA agarose was purchased from Novagen. All buffers were prepared from reagents of standard grade.

### Construction of plasmids and strains

*Expression and purification of MtBPL:* The BPL gene was amplified from *M.tuberculosis* chromosomal DNA by primers designed with restriction sites NdeI/HindIII. The PCR primers used for amplification are *Mt*BPL F1: 5′- GGAATTCCATATGGTGACCGACCGCGAT -3′; *Mt*BPL B1: 5′-CCCAAGCTTTTAACGCAAATGCAC-3′. The putative clone of BPL was sequenced using T7 promoter and terminator. It was cloned into pET 28a expression vector for recombinant expression in *E.coli* BL21 with hexahistidine tag at N-terminus. For optimal yield of recombinant protein, cells were grown at 37°C to an O.D_600 ∼_ 0.7 and induced with 0.1 mM isopropyl-1-thio-β-D-galactopyranoside (IPTG) for 4 h at 25°C. The cells were suspended in binding buffer (10 mM Tris-HCl, 500 mM NaCl, pH- 8.0), sonicated twice for 1 min on ice. The cell free sonicate was loaded onto charged Ni- NTA affinity resin (Novagen) and eluted with 100 mM imidazole. The eluted protein was dialyzed against standard buffer (10 mM Tris, 200 mM KCl, 2.5 mM MgCl_2_, pH-8.0). *Mt*BPL was a monomer (M_r_ ∼29,500) in native form as determined by gel exclusion chromatography (Superdex S200) on fast pressure liquid chromatography and Dynamic Light Scattering. The purity of the protein thus purified was in the range of 95–98% as determined by SDS-PAGE.

*Design of primers, expression and purification of BCCP:* The BCCP of *M.tuberculosis* has not been annotated as an individual gene but was part of acetyl CoA carboxylase in NCBI database. Thus BCCP of *M.tuberculosis* was cloned on the basis of the similarity to *E.coli* BCCP which was annotated as a protein of 156 amino acids with lysine residue at the 35^th^ position from C-terminal. The BCCP gene was cloned as a 468 bp PCR product amplified from *M.tuberculosis* chromosomal DNA as template using the primers *Mt*BCCP F1: 5′-CGCGGATCCATGCGCGGCGGGGGCCATATG-3′ and *Mt*BCCPF1: 5′CGCTCGAGCTAGTCCTTGATCCTCGCCAGTACC-3′.This PCR product was cloned at BamHI/XhoI sites of pET28a and the protein served as substrate for BPL. The cloned BCCP was sequence verified with T7 promoter and terminator using sequencer DNA star, Wisconsin. For optimal protein expression, cells were grown overnight in LB containing 30 µg/ml of kanamycin at 37°C for 14 h. Next day, 15% inoculum of this culture was added to fresh LB and grown for 1 h at 37°C. The culture was induced with 0.1 mM IPTG for 1 h at 25°C to prevent endogenous biotinylation by host BPL. The soluble protein did not bind to Ni- NTA agarose, probably because hexahistidine tag was masked. Hence, cells were purified under denaturation conditions (10 mM Tris, 100 mM NaH_2_PO_4_, 10 mM imidazole, 8 M Urea, pH- 8.0) and sonicated for 2 min, twice on ice. The cleared sonicate was loaded onto to a charged NiNTA column and the protein was eluted with elution buffer (10 mM Tris, 100 mM NaH_2_PO_4_, 10 mM imidazole, 8 M urea, pH- 4.5).The protein was further dialyzed against 10 mM Tris-HCl, pH- 7.5 containing decreasing concentration of urea.

### Determination of protein concentration

Concentration of protein was determined by Lowry's method and ultraviolet absorbance using an extinction coefficient of 1.26 mL mg^−1^ cm^−1^ for BPL and 1.373 mL mg^−1^cm^−1^ for BCCP. Both BPL and BCCP were exhaustively dialyzed against standard buffer of 10 mM Tris-HCl (pH- 7.5), containing 200 mM KCl and 2.5 mM MgCl_2_.

### Synthesis of Bio-5′-AMP

Bio-5′- AMP was synthesized by a modified method of Lane *et al* (34): To biotin (244 mg) dissolved in 4 ml of 75% aqueous pyridine with gentle heating 347 mg of ATP in 3 ml of 75% aqueous pyridine was added. To the reaction mixture, pre cooled 4.5 g of dicyclohexyl carbodiimide in 5 ml of absolute pyridine was added and reaction was carried out for 8 h at 0°C with constant stirring. The reaction mixture was kept aside at 4°C for 24 h to precipitate DCU which was removed by passing through a sintered glass filter. Bio-5′-AMP was separated from unreacted biotin and ATP by RP-HPLC performed on Waters equipment consisting of a Waters 2487 Dual Absorbance Detector. Analyses were carried out on an analytical column (Nucleosil 100 Å 5 µm C18 particles, 250×4.6 mm) using following solvent system: solvent A, 0.1% TFA in water; solvent B, 0.1% TFA in acetonitrile; flow rate, 1.0 mL min^−1^ with UV monitoring at 214 nm and 254 nm. Preparative column (Delta-PakTM 300Å 15 µm C18 particles, 200×2.5 mm) was used with an identical solvent system at a flow rate of 25 ml min^−1^. Mass spectra were obtained by electron spray ionization (ESI-MS).

### Analytical gel filtration chromatography

The oligomeric status of purified BPL was determined by gel filtration on Superdex S200 column and a fast protein liquid chromatography system (Pharmacia Biotech Inc). The column dimension was 30 cm×2.3 cm. The mobile phase was 50 mM Tris-HCl buffer, pH- 7.5, and flow rate was 0.2 ml/min. The molecular mass of standard proteins in kDa 150, 66, 45, 29 & 14 were, respectively, alcohol dehydrogenase, BSA, ovalbumin, carbonic anhydrase and lysozyme. The eluted proteins were detected by their absorbance at 280 nm.

The enzymatic reaction was carried out with 100 µM BPL, 3 mM ATP and 500 µM biotin, 5.5 mM MgCl_2_ at 37°C for 30 min and 300 µl of reaction mixture was loaded onto the column. The void volume (V_o_) was determined by calibrating column with blue dextran. From elution volume of standard proteins, molecular size of unknown protein, BPL and its enzymatic reaction mixture were determined.

*Computational modeling of ligand docking to MtBPL:* The structure of *Mt*BPL was modeled using SWISS-PROT software with *Mycobacterium tuberculosis* Biotin Protein Ligase as the template (Code: 2cghB) as the template (Ma Q. and Wilmanns M. unpublished). The ligands: biotin, desthiobiotin and bio-5′AMP were built using MOE (Molecular Operating Environment) and energy minimized under the MMFF94×forcefield. The docking of the ligands to *Mt*BPL was also performed with MOE using AMBER99 forcefield and the docked complex with least energy was chosen. The H bond interaction between the ligand atoms and amino acid residues of *Mt*BPL was obtained by LPC/CSU software (35).

*Assay of BPL*: BPL activity was assayed by measuring incorporation of [^14^C]- biotin (GE Healthcare, UK) into BCCP 156 as described by Chapman-Smith *et al* (10,11). The reaction contained 10 mM Tris-HCl, 200 mM KCl, 3 mM ATP, 5.5 mM MgCl_2_, 10 µM biotin and 1 µM of [^14^C] biotin (sp.activity 58.0 mCi/mmol), 0.1 mM DTT, 0.1 mg bovine serum albumin and 15 µM BCCP. The reaction was initiated by addition of 10 nM of BPL and incubated at 37°C for 30 min. The reaction mixture was added to biotin and trichloroacetic acid treated filters. After air drying, the filters were washed twice with 10% ice-cold TCA, ethanol and air dried and acid insoluble radioactivity was measured. A unit of BPL activity was defined as amount of enzyme required to incorporate 1 nM of biotin per min. Values for K_m_ and V_max_ were determined by non-linear least squares fit of the plots of substrate concentration *versus* velocity of reaction to *Michaelis-Menten* equation.

### Determination of thermodynamic parameters by Isothermal calorimetry

A MicroCal VP-ITC was used to determine binding parameters of biotin and desthiobiotin to BPL. All protein samples were dialyzed exhaustively against standard buffer, filtered and degassed before titration. Each calorimetric experiment was performed at 20°C; 10 µM of enzyme in reaction cell was titrated against 100 µM biotin, desthiobiotin, ATP, ADP, AMP from the syringe. But for determination of binding constant for bio-5′-AMP, 5 µM BPL was used. All heats of binding were corrected for heat of dilution by subtraction of average heat associated with multiple injections of ligand performed after saturation of protein (12). The binding constant (K_b_), enthalpy (ΔH), and stoichiometry (n) of the reaction were obtained from this corrected data by non-linear least square analysis using Origin 7.0 software package. The Gibbs free energy (ΔG), and entropy (ΔS), were calculated using the equation
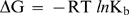
(1)

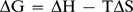
(2)


To determine association of biotin in presence of ATP, 10 µM of enzyme in standard buffer was allowed to equilibrate with 100 µM of biotin for 30 min in the reaction cell prior to titration with 100 µM of ATP.

### Fluorescence titration

Fluorescence titrations were made using a Fluorolog, tau-3 life-time spectrofluorimeter system according to the method of Xu *et al* (25). Measurements were performed in buffer containing 10 mM Tris-HCl, 200 mM KCl, 2.5 mM MgCl_2_, pH-7.5±0.02 at 20°C.The excitation wavelength was 295 nm, and emission was monitored from 310–450 nm. The excitation and emission slit widths were maintained at 4 nm. All measurements were made in ratio mode using rhodamine B as a standard. Titrations were performed by sequential addition of ligand to enzyme. The mixture was allowed to equilibrate for 2 min prior to measurement of spectra. The spectra were corrected for sample dilution and no correction was required for inner filter effect because absorbance of ATP at 295 nm at concentrations used was less than 0.005.

### Enzyme linked immunosorbent assay to determine specific incorporation of biotin

- To determine preferential utilization of substrate by BPL, a method akin to ELISA was used. The maxisorp plates (Nunc) were coated overnight with 15 µM BCCP in phosphate buffered saline (PBS) at 4°C.The wells were washed with PBS and dried off buffer. The BCCP was biotinylated at 37°C for 30 min and then streptavidin conjugated horseradish peroxidase (HRP) was incubated at 1∶5000 dilutions for 1 h at 37°C. The wells were washed and bound streptavidin was detected by TMB/H_2_O_2_ (Bangalore Genei, India).
- Competitive ELISA was performed to determine difference in interaction specificity of streptavidin for biotin/desthiobiotin using the ability of streptavidin to recognize biotin and desthiobiotin. In this assay, 100 µl of streptavidn HRP (1∶2500) was incubated with equal volume of varying concentration of biotin/desthiobiotin (5 µM–5 nM) for 1 h at 37°C. Then 100 µl of this solution was added to solid-phase biotinylated BCCP and incubated for 1 h at 37°C. Free unreacted streptavidin reacted with biotinylated BCCP. The bound streptavidin HRP was then detected by TMB/ H_2_O_2_.

*Avidin blot:* Biotinylation of BCCP by BPL was confirmed quantitatively by avidin blot. The reaction was carried out in a mixture containing 50 mM Tris, pH- 8.0, 3 mM ATP, 500 µM biotin, 5.5 mM MgCl_2_, 0.1 mM dithiothreitol, 0.1 mg/ml bovine serum albumin,15 µM BCCP. Reaction was initiated by 20 nM BPL at 37°C for 1 h. The reaction mixture was then resolved on 12% polyacrylamide gel and transferred to PVDF membrane. The non-specific sites on membrane were blocked with 1% BSA and then incubated with streptavidin conjugated to horseradish peroxidase (1∶2000) for 1 h at room temperature. The membrane was developed with AEC/ H_2_O_2_

## Supporting Information

Table S1 Structures of biotin, bio-5′AMP and desthiobiotin. The ligand atoms are numbered as per the LPC/CSU software.
(0.05 MB DOC)
