# Supplementary material for: Ligand Specificity of Group I Biotin Protein Ligase of Mycobacterium tuberculosis
Source: PLoS One. 2008 May 28;3(5):e2320. doi: 10.1371/journal.pone.0002320 (PMC2384007; doi:10.1371/journal.pone.0002320)
Supplement: Table S1 — Structures of biotin, bio-5′AMP and desthiobiotin. The ligand atoms are numbered as per the LPC/CSU software. (0.05 MB DOC) [file pone.0002320.s001.doc]

**Table S1: Structures of biotin, bio-5’AMP and desthiobiotin**

| **S.No.** | **Name** | **Structure** |
| --- | --- | --- |
| 1 | Biotin |  |
| 2 | Bio-5’-AMP |  |
| 3 | Desthiobiotin |  |

The ligand atoms are numbered as per the lpc/csu software.
